# Regulation of Mild Moxibustion on Uterine Vascular and Prostaglandin Contents in Primary Dysmenorrhea Rat Model

**DOI:** 10.1155/2021/9949642

**Published:** 2021-07-10

**Authors:** Xuemei Li, Sha Guo, Zhaoheng Chen, Kuiyu Ren, Hong Zhang, Shuguang Yu, Sha Yang

**Affiliations:** ^1^Acupuncture and Tuina School/The 3rd Teaching Hospital, Chengdu University of Traditional Chinese Medicine, Chengdu, Sichuan, China; ^2^Chengdu Pidu District Hospital of Traditional Chinese Medicine, Chengdu, Sichuan, China

## Abstract

**Objective:**

Primary dysmenorrhea (PD) is a common and high incidence disease in gynecology, which seriously affects the quality of life in young women. Our previous study found that mild moxibustion could treat abdominal pain of PD patients, but the mechanism is still unclear. Therefore, this study aims to partly investigate the treatment mechanism of moxibustion for PD, especially on uterine microcirculation.

**Methods:**

Forty 3-month-old Sprague Dawley female rats were randomly divided into four groups, including group A (saline control group, *n* = 10), group B (control plus moxibustion group, *n* = 10), group C (PD model group, *n* = 10), group D (PD. model plus moxibustion group, *n* = 10). The PD rat model was established by injecting estradiol benzoate and oxytocin. Mild moxibustion on Sanyinjiao (SP6) and Guanyuan (CV4) acupoints was once a day, 20 minutes per time, for 10 consecutive days. A vaginal smear was used to test the estrous cycle of rats. Uterine microvascular thickness was observed by stereomicroscope. And we detected the content of prostaglandin F_2*α*_ (PGF_2*α*_) and prostaglandin E_2_ (PGE_2_) in uterine tissue by enzyme-linked immunosorbent assay.

**Results:**

Mild moxibustion can enlarge the microvessels, improve the microcirculation disturbance, and relieve the swelling of the uterus in PD rats. During the mild moxibustion intervention, the contents of PGF_2*α*_ and PGE_2_ in uterus issues were synchronous increases or decreases and the changes of PGE_2_ were more obvious, but the changes of uterine microvasculature and morphology caused by the decrease of PGF_2*α*_ were greater than PGE_2_.

**Conclusion:**

Mild moxibustion at SP6 and CV4 acupoints may relax uterine microvascular obstacle by reducing the content of PGF_2*α*_ in uterine tissue, improve the microcirculation disorder, and then alleviate the PD rat's uterine swelling.

## 1. Introduction

Primary dysmenorrhea (PD) is defined as pain occurring with menses in the absence of pelvic pathology [1, 2]. Patients may suffer lower backache associated with painful menstruation, a condition more pronounced in women whose uterus is in the retroflexed position [2, 3]. The pain typically lasts for 8–72 h and is most severe during the first or second day of menstruation [3, 4]. PD is always accompanied by symptoms such as nausea, vomiting, diarrhea, fatigue, and insomnia. There is considerable variation in the prevalence of dysmenorrhea, depending on the definition used: 45–72 percent of all women and 43–93 percent of adolescent girls experience the condition [2]. Women with PD have lower quality of life (QOL) score because of recurrent abdominal pain, general health condition, and physical and social dysfunctions [5–7]. At present, it is treated with nonsteroidal anti-inflammatory drugs (NSAIDs) or oral contraceptive pills (OCPs), both of which work by reducing myometrial activity. However, these treatments are accompanied by renal and gastrointestinal side effects [3, 4]. Our goal of treatment is to provide adequate pain relief and reduction of symptoms with the least adverse effects [8].

Moxibustion is a traditional method of burning moxa sticks (usually made from herbal preparations containing *Artemisia vulgaris*) near an acupoint to cause a warm and painless sensation [9]. Moxibustion showed desirable merits in managing menstrual pain [10–16], given their treatment effects and economic costs. However, the mechanisms of action of moxibustion therapy are still largely unknown [17]. The factors are likely to be concluded as follows: temperature, infrared radiation, smoke, odor, and the type of moxa [18]. Many clinical and experimental studies have shown that moxibustion stimulation applied to the SP6 or CV4 acupoints (Sanyinjiao and Guanyuan moxibustion point) performed well in the treatment of pain induced of primary dysmenorrhea [10, 19–23]. SP6 and CV4 points are common compatibility schemes for the treatment of PD [24, 25]. The supporting degree of acupoint selection and compatibility is as high as 53.33%. Abaraogu et al. [26] found that pressing the SP6 acupoint can effectively relieve PD pain and reduce anxiety. However, the curative mechanisms of moxibustion remain poorly understood, which limits the application of moxibustion on a larger scale.

The pathogenesis of PD is still unclear, including abnormal uterine contraction, endocrine factors, nerve and neurotransmitter, and mental factors [27]. Uterine microcirculation disturbance is one of the main pathogenesis of dysmenorrhea [28]. The pathophysiology of primary dysmenorrhea is likely a result of the cyclooxygenase pathway producing increased prostanoids, particularly prostaglandins (PGs) [27]. Prostaglandin content plays an important role in the pathogenesis of PD. Further literature studies have found that excessive secretion of prostaglandins in the uterus was considered to be one of the main causes of dysmenorrhea pain [1, 29, 30]. The content of PGs in the endometrium of dysmenorrheal patients is higher than healthy women, and PGF_2*α*_/PGE_2_ is significantly greater as well. Fajrin et al. found that the greater the pain intensity score, the higher the levels of PGF_2*α*_ [31]. The increased production or unbalanced levels of PGs (PGF_2a_ and PGE_2_) create pain due to increased uterine contractility, decreased uterine blood flow, and increased sensitivity of peripheral nerves [32]. The uterine cavity pressure increases and the uterus wall blood flow reduces, so the microcirculation gets disordered and finally causes dysmenorrhea [33, 34]. Li et al.'s study [35] has shown that immediate acupuncture of SP6 may improve the uterine microcirculation disturbance through the nerve reflex pathway to the uterus, affect the synthesis and release of prostaglandins in the endometrium, and realize the analgesic effect. A previous study [36] has also shown that acupoint catgut embedding SP6 and CV4 can regulate the level of PGF_2a_ to treat PD.

At present, the research about moxibustion on PD in traditional Chinese medicine focuses on the regulation of endocrine hormones, immune function, nerve-related factors, improvement of uterine microcirculation, and other aspects. Our previous study demonstrated that moxibustion at the SP6 and CV4 acupoints can regulate the high resistance and low flow state of uterine microcirculation in PD patients to relieve pain [20]. It is not clear whether moxibustion can improve the microcirculation and shape of the uterus to relieve pain by regulating the balance of PGF_2*α*_/PGE_2_. Hence, this study aimed to investigate the effect and mechanism of moxibustion on SP6 and CV4 acupoints on the release of PGF_2*α*_/PGE_2_ and the regulation of uterine microcirculation.

## 2. Materials and Methods

### 2.1. Ethical Statement

Animal care and experimental procedures used in the current study were filed and supervised by the animal experimental center of Chengdu University of traditional Chinese medicine (ethics no. 2019-13). This study was carried out adhering to guidelines provided by the National Institutes of Health for the Care and Use of Laboratory Animals and all efforts were made to minimize the suffering of animals.

### 2.2. Experimental Animals

Adult female Sprague Dawley (SD) rats weighing 240 ± 20 g were purchased from the Chengdu Dashuo experimental animal Co. (medical laboratory animal certificate number: SCXK (Chengdu) 2015-030). All the rats were kept in the Experimental Animal Center of Chengdu University of Traditional Chinese Medicine. The animal room was under controlled conditions (temperature, humidity, and a 12-hour light-dark cycle).

### 2.3. Construction of PD Rats

**Figure 1:**
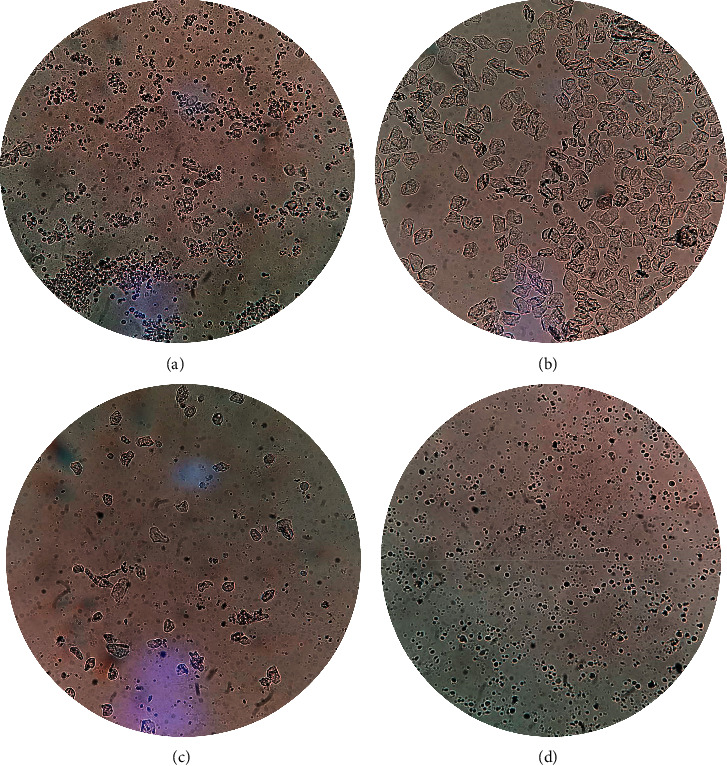
The regular vaginal smear of the typical rat estrus cycle. The cells of the different estrous cycles in rats were as follows: the small and round nucleated epithelial cells were the main cells in the prophase of estrus. In estrus, irregular squamous epithelial cells predominate. White blood cells, nucleated epithelial cells, and keratinized epithelial cells can be seen in the later stage of estrus, but the total number is small. During the estrus, most of them were white cells, and a few of them were nuclear epithelial cells and keratinized epithelial cells. (a) Propstrum. (b) Estrus. (c) Postestrus. (d) Diestrus.

**Figure 2:**
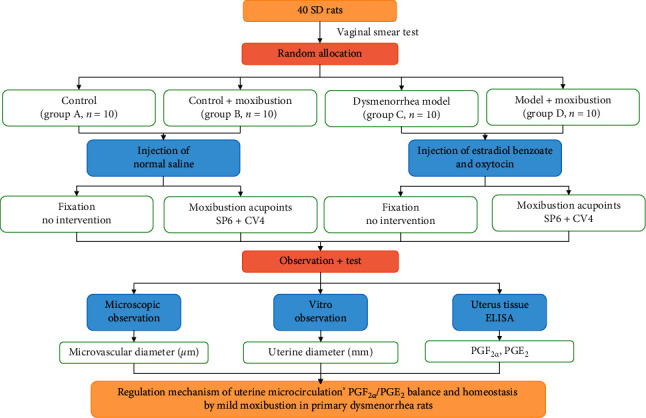
Flowchart of experiment development. SP6 = Sanyinjiao; CV4 = Guanyuan.

Forty female SD rats were fed adaptively for 7 days, and a vaginal smear was used to screen the estrous cycles of rats for 7 days. During the experiment, the typical four cycles of vaginal smear were observed (Figure 1). Rats with regular estrous cycles were selected for this study. According to the random number table, they were divided into four groups, including group A (control group), group B (control plus moxibustion group), group C (PD model group), group D (PD model plus moxibustion group). The rats of groups C and D were injected with estradiol benzoate subcutaneously and intraperitoneal oxytocin to establish the PD model [37]. The rats were in the estrus period and began to inject estradiol benzoate. The rats were injected subcutaneously with estradiol benzoate injection for ten days (0.5 mg/day on the 1^st^ and 10^th^ days, 0.2 mg/day on the 2^nd^–9^th^ days), and 2 u per rat intraperitoneal injection of oxytocin 1 hr after the last injection of estradiol benzoate. Group A and group B rats were injected subcutaneously with saline of the same volume as estradiol benzoate on the 1^st^–10^th^ days. One hour after the last injection, they were injected with the same volume saline of oxytocin. Group A and group B were injected subcutaneously with the same volume of saline as estradiol benzoate on the 1^st^–10^th^ days. One hour after subcutaneous injection of saline on the 10^th^ day, the rats were injected intraperitoneally with the same volume of 2 u saline as oxytocin [33, 35]. A flowchart of experiment development is shown in Figure 2.

### 2.4. Evaluation of PD Model

After the completion of the model building, observed the writhing times of every rat within 20 minutes. The model rats showed twisting responses (abdominal contraction, concave, trunk, and hind limbs extension, one limb rotation, uterine contraction), indicating that the PD rats model was successfully prepared.

### 2.5. Treatment of Moxibustion

**Figure 3:**
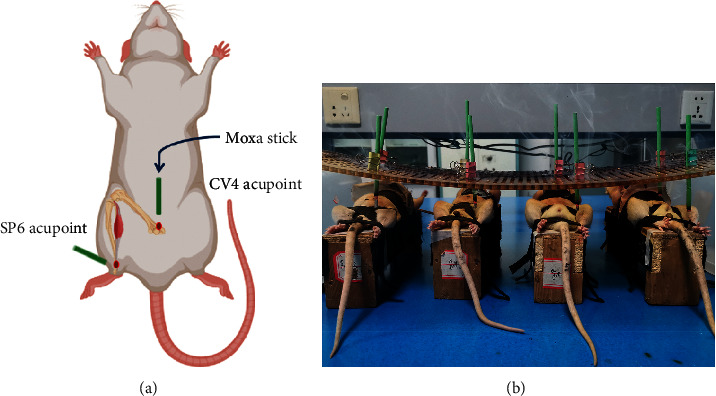
(a, b) The location of SP6 and CV4 acupoints on the body of SD rats. SP6 is located 10 mm above the tip of the inner ankle of the hind limb, and CV4 is located about 25 mm below the umbilicus.

Groups A and C were put on the self-made rat clothes and fixed on the frame to expose their abdomen and leg and received no treatment (Figures 3(a) and 3(b)). Groups B and D received moxibustion on “SP6” and “CV4” from the 1^st^ to 10^th^ days after model establishment. First, we put on the self-made rat clothes and fixed them on the frame to expose their abdomen and leg. The locations of SP6 and CV4 acupoints were shown in Figure 3(a). Shave the hair of acupoints by 1 × 1 cm, mark the acupoint position with a marking pen, and light the moxa stick and placed it 2-3 cm away from the skin of the rat. Then, make moxibustion of the above two acupoints gently, and replace the moxa stick when it was close to burnout, once a day, each time for 20 min [38], for 10 days [12] (choose the right SP6 for 1–5 d and the opposite side for 6–10 d). All manipulations were performed between 8:00 a.m. and 12:00 a.m. every day to minimize the influence of circadian rhythms.

### 2.6. Observed the Number and Shape of Microvasculature under the Microscope

After the rats were anesthetized with 0.2 ml/100 g of 1.5% pentobarbital sodium, we made an opening about 2-3 cm long at about 0.5 cm outside the midline of the abdomen and then pulled out one side of the uterus and its ligament. We selected the middle section of the uterus and put it under the stereomicroscope. We observed the color and number of uterus microvasculature under the stereomicroscope (40X) and took pictures. At last, we calculated the microvascular diameters (*μ*m) of pictures by Image Proplus Software.

### 2.7. Measurement of Uterine Thickness In Vitro

After the observation of uterine microvasculature under the stereomicroscope, the rats were further killed by spinal dislocation. The uterus was quickly and completely removed (in a “V” shape). The uterine tissue was placed in a culture dish (the culture dish was placed above the ice plate), and the connective tissue and fat attached to the uterine wall were carefully removed (attention should be paid to not retaining the ovaries at the distal end of the bilateral uterus). In the process of stripping, it is necessary to avoid puncturing the uterine body due to instrument operation and ensure the liquid filling in the uterine body. The thickness (mm) of the isolated uterus was measured at the midpoint between the distal end of the uterus and the uterine horn. After the measurement, the uterus was stored in Eppendorf (EP) tube and transferred to liquid nitrogen. All uteruses were stored at −80°C.

### 2.8. Detection of the Content of PGF_2*α*_ and PGE_2_ in Tissue by Enzyme-Linked Immunosorbent Assay

**Figure 4:**
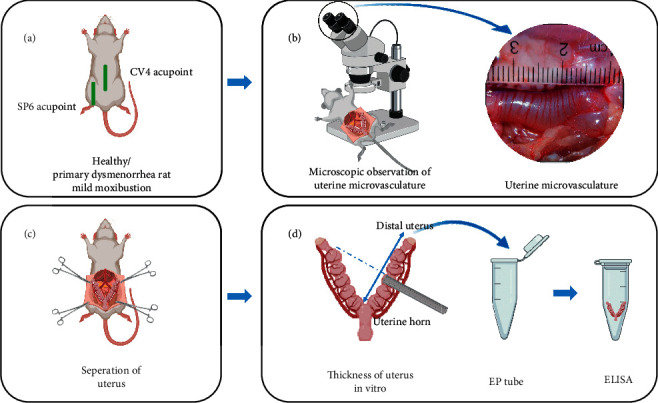
The schematic diagram of the whole experimental process of moxibustion intervention on healthy and PD rats. (A) The moxibustion on health and PD rats. (B) Microscopical observation of uterine microvasculature in rats. (C) The separation of the uterus. (D) Measurement of uterine thickness in vitro and detection PGF_2*α*_ and PGE_2_ of uterus tissue by ELISA.

We took 100 mg of frozen uterine tissue from the ipsilateral segment and ground it with a 9-fold homogenate medium. Then, the grinding liquid was centrifuged at 3000–4000 r for 10 min. The supernatant was taken and prepared into 10% tissue homogenate, which was placed in a refrigerator at 4°C for testing. Diluent and standard liquid were prepared according to the instructions. The samples were incubated at room temperature for 1.5 hours and then washed. Antibodies, enzymes, signal enhancers, and enzymes were successively added for incubation and washing. Finally, after adding the substrate color development and termination solution, the absorbance values of each hole were detected at 450 nm. The standard curve was drawn with the absorbance value of the standard hole and its corresponding concentration, and the contents of PGF_2a_ and PGE_2_ in the uterine tissues of the rat samples were calculated. The contents of PGE_2a_ and PGF_2_ were determined by enzyme-linked immunosorbent assay (ELISA) and operated strictly according to the kit instructions (the detection kit brand is Elabscience).  Figure 4 shows the schematic diagram of the whole experimental process of moxibustion intervention on healthy and PD rats.

### 2.9. Statistical Analysis

GraphPad Prism 8.0 software was used for statistical analysis. The normal data were expressed as mean ± standard deviation (SEM) and the nonnormal distribution data as median (lower quartile, upper quartile). The data of each group conform to a normal distribution. Two-way ANOVA was used for the comparison among the groups, and the Turkey test was used for the multiple comparison after the event. *P* < 0.05 was considered as the difference was significant.

## 3. Results

### 3.1. Difference of Writhing Times after Molding

Because groups A and B did not use estradiol benzoate and oxytocin to induce pain, there was no writhing reaction within 20 minutes. After modeling, groups C and D showed a significant writhing response (Table 1). For groups C and D, the writhing latency was shortened. The results showed that the model of estradiol benzoate combined with oxytocin was successful, and the baseline of groups C and D was comparable.

### 3.2. Effect of Moxibustion on the General State of Rats

The results showed that moxibustion at the SP6 and CV4 acupoints for ten consecutive days can regulate the general state of rats. The hair color of rats in group A was glossy and they moved freely. In group C, the hair color was haggard. And they depilated with obvious grasping stress. In group D, the overall state of rats was better than that of group C, and the hair color was glossy and active.

### 3.3. Effect of Moxibustion on the Thickness of Uterus In Vitro (mm)

A previous study by Dmitrovic et al. found that the uterus of dysmenorrhea women was larger than that of healthy women, especially in the transverse, anterior, and posterior diameters [39]. We wanted to know the relationship between uterine morphology, uterine microvasculature, and uterine prostaglandin. Therefore, we dissected the uterine tissue and measured its thickness. The two main effects (independent variables) described below refer to the body state of rats (primary dysmenorrhea model rats/healthy rats) and moxibustion or not.

**Figure 5:**
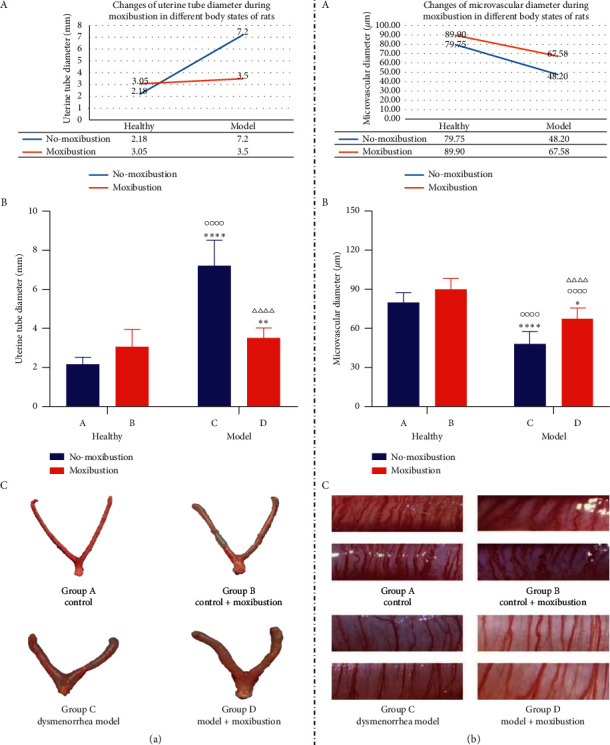
In (a), changes of uterine tube diameter during moxibustion in different body states of rats (A), thickness of uterus in vitro (mm) (B), and four groups of the typical uterus in vitro (C) are shown. In (b), changes of microvascular diameter during moxibustion in different body states of rats (A), effects of moxibustion on uterine microvascular diameter under microscope (B), and microscopic view of uterine microvascular (C) are shown. Data are expressed as the upper quartile and the lower quartile. ^*∗*^*P* < 0.05, ^∗∗^*P* < 0.01, ^∗∗∗∗^*P* < 0.0001 versus group A. ^οοοο^*P* < 0.0001 versus group B. ^ΔΔΔΔ^*P* < 0.0001 versus group C.

In terms of the thickness of the isolated uterus, the two main effects were statistically significant (*P* < 0.0001, *P* < 0.0001), and there was a significant interaction between the two main effects (*P* < 0.0001). The effect of moxibustion on the thickness of the isolated uterus varies with the body state (A in Figure 5(a)).

Compared with group A, there was no statistical difference between group A and group B (*P* > 0.05), but the diameter of the uterus in vitro has a trend of increased in group B. In group C, the diameter of the uterus in vitro has increased significantly (*P* < 0.0001), and in group D, the diameter of the uterus in vitro increased (*P* < 0.001). Compared with group B, the diameter of the uterus in vitro in group C has increased significantly (*P* < 0.0001). The diameter of the uterus in vitro has a trend of increase in group D (*P* > 0.05), the difference was not statistically significant. Compared with group C, the results showed that moxibustion at the SP6 and CV4 acupoints for ten consecutive days significantly decreased the diameter of the uterus in vitro (*P* < 0.0001) (B and C in Figure 5(a)).

### 3.4. Effect of Moxibustion on Uterine Microvascular Diameter (*μ*m)

Microcirculation includes arterioles, posterior arterioles, true capillaries, and blood capillary. Uterine microvasculature is an important part of microcirculation. Some studies found that there are obvious microcirculation disorders in PD rats, such as the uneven thickness of uterine microvasculature and capillaries, the contraction of diameter [35], and the common fracture of capillaries, even the contraction to the smooth muscle layer of the uterus [40]. Acupuncture at CV4 and SP6 acupoints can improve microcirculation disorders. The related factors affecting uterine microcirculation include temperature and body fluid regulation. Therefore, this part of the study mainly observed the influence of moxibustion on SP6 and CV4 acupoints through observing uterine microvasculature in vivo.

In terms of microscopic microvascular, the main effects of the two factors were statistically significant (*P* < 0.0001, *P* < 0.0001), and there was no interaction between the two main effects (*P* > 0.05). The effect of moxibustion on uterine microvasculature is not different from the body state of rats (A in Figure 5(b)).

Compared with group A, the diameter of uterine microvasculature in group B tended to be coarser (*P* > 0.05). The microvascular diameter in group C decreased significantly (*P* < 0.0001), and that in group D decreased (*P* < 0.05). Compared with group B, the microvascular diameter of groups C and D decreased significantly (*P* < 0.0001), and the difference was statistically significant. Compared with group C, the microvascular diameter in group D was significantly thicker (*P* < 0.0001), and the difference was statistically significant (B and C in Figure 5(b)).

### 3.5. The Effect of Moxibustion Applied to the SP6 and CV4 Acupoints on the PGs (PGF_2*α*_ and PGE_2_) Levels

**Figure 6:**
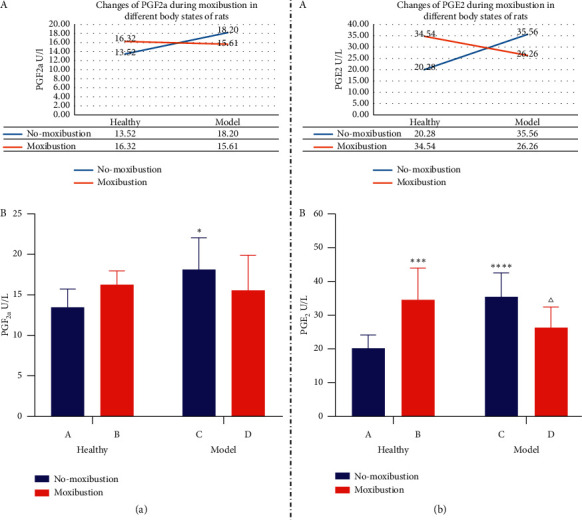
In (a), changes of PGF_2a_ during moxibustion in different body states of rats (A) and effects of moxibustion on the content of PGF_2a_ in uterine tissue (B) are shown. In (b), changes of PGE_2_ during moxibustion in different body states of rats (A) and effects of moxibustion on the content of PGE_2_ in uterine tissue (B) are shown. PGF_2a_ data are expressed as the mean ± SEM. ^*∗*^*P* < 0.05 versus group A. PGE_2_ data are expressed as the upper quartile and the lower quartile. ^∗∗∗^*P* < 0.001, ^∗∗∗∗^*P* < 0.0001 versus group A. ^Δ^*P* < 0.05 versus group C.

In terms of PGF_2a_, the main effect of the two independent variables was not statistically significant (*P* > 0.05), but the interaction effect was statistically significant (*P* < 0.05). The effect of moxibustion on PGF_2a_ in uterine tissue varies with different body states (A in Figure 6(a)). Compared with group A, the PGF_2*α*_ in groups B and D increased (*P* > 0.05). The PGF_2*α*_ in group C increased (*P* < 0.05), and the difference was statistically significant. Compared with group B, PGF_2*α*_ in group C increased (*P* > 0.05), while PGF_2*α*_ in group D decreased (*P* > 0.05). Compared with group C, PGF_2*α*_ in group D decreased (*P* > 0.05) (B in Figure 6(a)).

In terms of PGE_2_, the main effect of the two independent variables was not statistically significant (*P* > 0.05), but there was a significant interaction effect between the two variables (*P* < 0.0001). The effect of moxibustion on PGE_2_ in uterine tissue varies with different body states (A in Figure 6(b)). Compared with group A, the PGE_2_ in groups B and C increased (*P* < 0.001, *P* < 0.0001), and the difference was statistically significant. The PGE_2_ in group D increased (*P* > 0.05). Compared with group B, PGE_2_ in group C increased (*P* > 0.05), while PGE_2_ in group D decreased (*P* > 0.05). Compared with group C, PGE_2_ in group D decreased (*P* < 0.05) (B in Figure 6(b)).

## 4. Conclusion

According to Tables 2 and 3 and the above results, the following conclusions can be further deduced.

(1) After estradiol benzoate and oxytocin made the model, the uterine microvessels of rats became thinner, the isolated uterus became swollen and thicker, and the contents of PGF_2*α*_ and PGE_2_ increased.
(2) Moxibustion can thicken the uterine microvessels of PD rats, thin the isolated uterus, and reduce the contents of PGF_2a_ and PGE_2_.
(3) Moxibustion can thicken the uterine microvessels of healthy rats, thicken the isolated uterus, and increase the contents of PGF_2a_ and PGE_2_.
(4) Moxibustion has different regulatory effects on rats in different body states.
  ① The regulating effect of moxibustion on the microvessels of PD rats is greater than that of healthy rats.
  ② Moxibustion can regulate the prostaglandin index in two ways. Moxibustion decreased the contents of PGF_2a_ and PGE_2_ in PD rats and increased the contents of PGF_2a_ and PGE_2_ in healthy rats.
(5) Combined with the changes of microvascular effect in rats, although the content of PGE_2_ changed more obviously, the contractile effect of PGF_2a_ was dominant.
(6) In conclusion, moxibustion at SP6 and CV4 can reduce dysmenorrhea in PD rats, and its mechanism may be to reduce the content of PGF_2*α*_ and PGE_2_ in uterine tissue, relax uterine microvessels, improve microcirculation disorder, so as to reduce uterine swelling, and achieve the purpose of treating dysmenorrhea.

## 5. Discussion

The literature research shows that PGF_2*α*_ mainly plays a contractile role in the uterus, and PGE_2_ plays a diastolic role in the uterus. Dysmenorrhea is characterized by fluctuation and intermittence, with the most intense pain at the first 24–36 hours of menstruation, which is consistent with the maximum time of prostaglandin release into menstrual fluid [41]. The occurrence of PD pain is related to the production of large amounts of PGs in the uterus [1, 42–45], and the intensity of pain is directly proportional to the content of PGF_2*α*_ [31, 46]. The most basic structure and function unit of the uterine microcirculation system is the microcirculation between uterine microvasculature and capillary. PGF_2*α*_ can constrict the uterine microvascular [47], resulting in the decrease of the local blood flow of the uterus, the disturbance of the microcirculation of the uterus, the abnormal tissue fluid exchange caused by the long-term tissue ischemia and hypoxia, the morphological changes of the uterus, and finally a series of symptoms of PD.

This study found that the content of PGF_2*α*_ and PGE_2_ in uterine tissue increased in the intervention of moxibustion on healthy rats, and the microvasculature became coarser. After models were made by estradiol benzoate combined with oxytocin, the two kinds of prostaglandins in the uterus of PD rats increased, the content of PGE_2_ was higher than that of PGF_2*α*_, and the microvasculature was thinner, suggesting that the contraction effect of PGF_2*α*_ was stronger than the relaxation effect of PGE_2_. Moxibustion intervention in PD rats, the two prostaglandins in uterine tissue were reduced, the content of PGE_2_ was significantly lower than PGF_2*α*_, and the microvessels were significantly thicker, suggesting that the contraction effect of PGF_2*α*_ was more obvious than the relaxation effect of PGE_2_. To sum up, PGF_2*α*_ and PGE_2_ in the uterus of rats showed a homotropic change, and the change of PGE_2_ was more obvious, but PGF_2a_ played a leading role in contraction.

Combined with the literature research and this study (Table 1), it is speculated that the content of PGF_2*α*_ and the contraction of uterine microvasculature may cause local ischemia and anoxia, microcirculation disturbance, and the change of uterine tissue morphology and thickening. Moxibustion can reduce the content of PGF_2*α*_ and the relaxation of uterine microvasculature in PD rats, which can lead to the relief of local ischemia and hypoxia, the improvement of microcirculation disturbance, and the reduction of swelling of the uterus in vitro. To sum up, moxibustion of SP6 and CV4 points may relax the uterine microvasculature by reducing the content of PGF_2*α*_ in the uterine tissue, to improve the microcirculation of the uterus, then alleviate the degree of uterine swelling, and finally achieve the effect of relieving dysmenorrhea.

We regarded prostaglandins as the main entry point, combined with PGF_2a_/PGE_2_ and uterine microcirculation to explore the possible mechanism of moxibustion treat PDs. Many clinical and experimental studies have shown that moxibustion stimulation applied to the SP6 or CV4 acupoints (Sanyinjiao and Guanyuan moxibustion point) performed well in the treatment of pain induced of primary dysmenorrhea [10, 19–23]. SP6 and CV4 points are common compatibility schemes for the treatment of PD [24, 25]. SP6 can relieve the pain of PD immediately [44, 48, 49], especially for PD patients with cold and dampness stagnation pattern [50]. Moxibustion may regulate the content of prostaglandin in the uterus of PD through its light effect, heat effect, moxa fume, drug composition, and so forth. Further, improve the microcirculation of the uterus, finally, relieve the shape of the uterus, and achieve the effect of relieving dysmenorrhea [51].

In the whole process of the experiment, animal selection, model making, intervention, and efficacy evaluation were strictly controlled. Moxibustion significantly improved uterine microvascular and isolated uterine morphology, while the regulation of prostaglandin only showed a trend change. Combined with the particularity of moxibustion as a supplementary alternative therapy, the whole experimental process was reviewed and analyzed. The reasons considered may be related to the lack of moxibustion quantity and treatment course. Further research directions include but are not limited to the following. 1. In this study, moxibustion intervention on the prostaglandin of PD rats shows a trend change, which may be related to the course of treatment, cycle, and insufficient amount of moxibustion. In the further experiment, different control groups are set up to study the quantity effect relationship of moxibustion intervention on PD. 2. This experiment speculates that the therapeutic effect of moxibustion on PD rats is greater than that of healthy rats. We can set up the matching experiment to study the index change before and after the intervention and further clarify the specific effect difference of moxibustion on healthy and PD rats. 3. Moxibustion regulates the contraction of uterine microvascular by prostaglandin, causes the change of uterine morphology, and relieves the pain process. It is worth further exploring whether there is a new target in the information transduction process between prostaglandin and uterus. 4. There may be differences in the effects of different cycles of moxibustion on PD, which can be studied by incorporating rats in different emotional periods to determine the best intervention time of moxibustion for PD.

In conclusion, moxibustion of SP6 and CV4 acupoints may relax the uterine microvasculature by reducing the content of PGF_2*α*_ in the uterine tissue, to improve the microcirculation of the uterus, then alleviate the degree of PD rat's uterine swelling, and finally, achieve the effect of relieving dysmenorrhea. It will be further studied whether moxibustion can relieve dysmenorrhea symptoms through light effect, heat effect, or drug composition of moxa fume.

## Figures and Tables

**Table 1:** Writhing latency of rats after modeling.

Groups	Numbers	Writhing latency (min)	Writhing times
C	10	8.50 ± 6.45	11.30 ± 9.84
D	10	10.00 ± 5.98	8.30 ± 5.43

Data are expressed as the mean ± SEM.

**Table 2:** Index changes and interaction effects of moxibustion in different body states of rats.

Observation index	Source of difference	SS	MS	*F*	*P* value
*The thickness of uterus in vitro* (mm)	Row factor	74.80	74.80	102.23	*P* < 0.0001
	Column factor	20.02	20.02	27.36	*P* < 0.0001
	Interaction	52.21	52.21	71.36	*P* < 0.0001

Microvascular diameter (*μ*m)	Row factor	7253.87	7253.87	99.53	*P* < 0.0001
	Column factor	2180.05	2180.05	29.91	*P* < 0.0001
	Interaction	212.89	212.89	2.92	*P* > 0.05

*PGF*_2*a*_ (U/L)	Row factor	39.34	39.34	3.89	*P* > 0.05
	Column factor	0.11	0.11	0.01	*P* > 0.05
	Interaction	72.42	72.42	7.16	*P* > 0.05

*PGE*_2_ (U/L)	Row factor	122.49	122.49	2.72	*P* > 0.05
	Column factor	61.46	61.46	1.36	*P* > 0.05
	Interaction	1387.89	1387.89	30.80	*P* < 0.05

Row factor: different body states of rats (healthy/primary dysmenorrhea model); column factor: moxibustion or not; SS: sum of squares between groups; MS: mean square.

**Table 3:** Summary of the effects of moxibustion on the regulation of uterine microvascular in healthy and PD rats.

Intergroup comparison	The thickness of uterus in vitro (mm)	Microvasculature diameter (*μ*m)	PGF_2*α*_ (U/L)	PGE_2_ (U/L)
A-B	Thickening trend *P* > 0.05	Thickening trend *P* > 0.05	Rising trend *P* > 0.05	Rising *P* < 0.001
A-C	Obvious thickening *P* < 0.0001	Obvious thinning *P* < 0.0001	Rising *P* < 0.05	Rising *P* < 0.0001
C-D	Thinning *P* < 0.0001	Obvious thickening *P* < 0.0001	Downward trend *P* > 0.05	Downward *P* < 0.05
A-D	Thickening *P* < 0.001	Thinning *P* < 0.05	Rising trend *P* > 0.05	Rising trend *P* > 0.05
B-D	Thickening trend *P* > 0.05	Obvious thinning *P* < 0.0001	Downward trend *P* > 0.05	Downward trend *P* > 0.05

A = control group; B = control plus moxibustion group; C = PD model group; D = PD model plus moxibustion group.

## Data Availability

The data used to support the findings of this study are included within the article.
